# The formation of “mega‐flocks” depends on vegetation structure in montane coniferous forests of Taiwan

**DOI:** 10.1002/ece3.8608

**Published:** 2022-02-18

**Authors:** Chun‐Chieh Liao, Tzung‐Su Ding, Chao‐Chieh Chen

**Affiliations:** ^1^ School of Forestry and Resource Conservation National Taiwan University Taipei Taiwan; ^2^ Division of Ecology and Evolution Research School of Biology Australian National University Canberra Australian Capital Territory Australia; ^3^ The Experimental Forest College of Bio‐resources and Agriculture National Taiwan University Nantou Taiwan; ^4^ 38023 Department of Biomedical Science and Environmental Biology Kaohsiung Medical University Kaohsiung Taiwan

**Keywords:** avian assemblage, coniferous forest, mixed‐species bird flock, structural heterogeneity, vegetation succession

## Abstract

A mixed‐species bird flock is a social assemblage where two or more bird species are moving together while foraging and might benefit from increased foraging efficiency and antipredator vigilance. A “mega‐flock,” which includes flocking species from different vegetation strata, often exhibits high species diversity. Mechanisms for the formation of mega‐flocks have not yet been explored. In this study, we evaluated the influence of vegetation structure and bird species diversity in driving the occurrence of mega‐flocks. We investigated the composition of mixed‐species flocks, local bird communities, and vegetation structure in five vegetation types of two high‐elevation sites in central Taiwan. Mega‐flocks occurred more frequently in pine woodland than later successional stages of coniferous forests. However, species richness/diversity of local bird communities increased along successional stages. Therefore, vegetation variables exhibit more influence on the occurrence of mega‐flocks than local bird communities. Besides foliage height diversity, understory coverage also showed positive effects on flock size of mixed‐species flocks. Our results indicated that pine woodlands with more evenly distributed vegetation layers could facilitate the interactions of canopy and understory flocks and increase the formation of mega‐flocks and thus the complexity of mixed‐species flocks.

## Graphical abstract

Mega‐flocks occurred most frequently in pine woodland and decreased in later successional stages of coniferous forests in alpine Taiwan. However, species diversity of local bird communities increased along successional stages. Therefore, vegetation variables exhibit more influence on the occurrence of mega‐flocks than local bird communities.

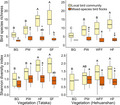

## 1 INTRODUCTION

A group of two or more bird species that forage together and move concordantly is known as a mixed‐species bird flock (Hutto, 1987; Morse, 1970; Moynihan, 1962). Such flocks occur in most biogeographic regions, elevational ranges, and habitat types (Greenberg, 2000; Powell, 1985; Zou et al., 2018). It has been reported that 19% of the world's bird species participate in terrestrial mixed‐species flocks, representing a crucial component of the global avifauna (Zou et al., 2018). Of various habitat types, forests are the prime habitat for this flocking phenomenon, and a high proportion of recent studies on mixed‐species bird flocks come from forests (Colorado Z. & Rodewald, 2015; Goodale et al., 2014; Jones & Robinson, 2020; Mokross et al., 2014; Zhang et al., 2013). In forest ecosystems, mixed‐species flocking positively enhances the survival and fitness of participants through greater foraging success (Sridhar & Shanker, 2014; Srinivasan, 2019), higher antipredator vigilance (Magrath et al., 2015), and might allow local birds to persist in a challenging environment (Mammides et al., 2015; Martinez et al., 2018). In addition, flock characteristics (e.g., species richness, flock size, and flocking frequency) have been considered as important metrics to signify environmental changes in forest habitats (Goodale et al., 2014; Jones & Robinson, 2020; Maldonado‐Coelho & Marini, 2004; McDermott & Rodewald, 2014; Zhang et al., 2013).

Vegetation complexity has long been known as a significant component affecting local bird communities, including species richness, abundance, and diversity (Ding et al., 2008; Lin et al., 2019; MacArthur & MacArthur, 1961; Schieck & Song, 2006; Zhang et al., 2011). The more diverse the microhabitats in a forest are, the greater are the ecological niches that become available, and the greater are the number of species that are expected to co‐exist (Keller et al., 2003; MacArthur et al., 1966). Recently, some studies indicated that flock characteristics are highly sensitive to habitat changes, including forest fragmentation (Jones & Robinson, 2020; Maldonado‐Coelho & Marini, 2004; Mokross et al., 2014; Sridhar & Sankar, 2008) and natural succession of forest gradients (Mokross et al., 2018; Zhang et al., 2013). For example, flock diversity changes along successional gradients, and a greater number of mixed‐species flocks and greater species richness are recorded in forests of middle and later successional stages (Montaño‐Centellas & Jones, 2021; Zhang et al., 2013). Additionally, forests with more complex vegetation structure are usually associated with higher species richness per flock and larger flock size (Colorado Z. & Rodewald, 2015; Lee et al., 2005; Mokross et al., 2014). Therefore, the overall vegetation structure might affect the size of the bird community, and also the number of species available to join mixed flocks (open membership hypothesis; according to Montaño‐Centellas & Jones, 2021).

In lowland tropical rainforests, where vegetation structure has multiple layers and a relatively high canopy, canopy, and understory bird species often form separate flocks (Munn, 1985; Powell, 1985; Zou et al., 2011). When these two flock types meet, they often mix and behave like a single flock (Munn, 1985). Munn (1985) reported that some canopy flocks spend several hours each day associating with understory flocks, and occasionally nonpermanent tanager‐honeycreeper flocks also join the combined flocks. Such combined flocks could consist of 60 to 70 bird species and close to 100 individuals. Also, in a lowland tropical forest of northeastern India, three sympatric but distinct flock types co‐exist, including canopy, understory, and “large‐bodied” flocks (Srinivasan et al., 2012). These flocks differ in participants’ body mass and vertical stratum use. Occasionally, birds from the different flock types come together to form a single mixed‐species flock (Srinivasan et al., 2012). Greenberg (2000) named this type of flock a “mega‐flock,” which usually includes flocking species from the canopy, mid‐story, and understory layers and contains a remarkably high species diversity and flock size (Munn, 1985; Musschenga, 2012; Poulsen, 1996).

On the other hand, Poulsen (1996) found that mixed flocks in a high‐altitude secondary forest in the Andes of Ecuador comprised members from all vertical levels. These mega‐flocks mainly formed in areas with low stature of trees and open structure of the canopy (Poulsen, 1996). This study suggested that the compacted vegetation structure in high‐altitude secondary forests might contribute to the formation of multi‐stratum flocks, because the costs of flock‐merging might be greatly reduced in secondary forests compared to lowland rainforests. At the same time, vegetation structure may affect the costs of flock joining for the participating species. Although the above studies have indicated that vegetation complexity is an important factor influencing different aspects of mixed‐species flocking, the relationships among mixed‐species flocks, the local bird community, and the vegetation structure are still poorly understood (Zou et al., 2018).

Mixed‐species flocks are mainly composed of insectivorous species that search on foliage for visible prey, and similar species are more likely to flock together (Goodale et al., 2020; Jones et al., 2020). Therefore, attendant species that take concealed prey might have to sacrifice their foraging efficiency in order to join the moving flocks (Darrah & Smith, 2013). In some cases, attendant species modify their foraging strategy for keeping up with a mixed flock (Hino, 1998; Pomara et al., 2003; Zou et al., 2011). Similarly, for canopy attendant species to join understory flocks or vice versa, some costs associated with energy investment and foraging efficiency must be considered (Darrah & Smith, 2013; Hutto, 1988). Therefore, if the costs to join flocks of different strata are reduced to a limited amount, the formation of such multi‐stratum flocks would become likely to occur.

To our knowledge, mega‐flocks (mixing of different types of flocks) are commonly found in tropical forests (Greenberg, 1984; Munn, 1985; Poulsen, 1996; Powell, 1985), but are rarely reported from temperate or subtropical regions. In the high‐elevation mountain areas of Taiwan Island, both Palaearctic bird species (e.g., tit, kinglet, and nuthatch) and oriental bird species (e.g., babbler, fulvetta, and laughingthrush) are present sympatrically. Canopy flocks and understory flocks have been occasionally observed foraging together in the coniferous mountain forests of Taiwan (Liao, 2015). These biomes are an excellent site for studying mega‐flocks because vegetation types of different dominant species, vertical foliage structure, and canopy height are available in a relatively confined area. In the present study, five different successional stages (bamboo grassland, pine woodland, white fir forest, hemlock forest, and spruce forest) were selected to examine the influence of vegetation structure on the occurrence of mega‐flocks.

The specific objectives of this study were to: (1) determine whether the occurrence of mega‐flocks change with the successional stage of the forest; (2) discriminate the influence of local bird communities from vegetation attributes on mega‐flocks; and (3) pinpoint any specific structural components that might promote the formation of mega‐flocks. Since the diversity of mixed flocks was found to increase with forest successional stages (Jones & Robinson, 2020; Zhang et al., 2013), we expect that a difference in mega‐flock metrics could be detected among different habitat types and the responsible key variables could be identified. To our knowledge, this is the first study to explore the influence of vegetation structure on the formation of mega‐flocks. Previous studies on mega‐flocks were predominantly descriptive (Munn, 1985; Poulsen, 1996), and the mechanisms for the formation of mega‐flocks were not studied as yet.

## 2 METHODS

### 2.1 Study sites

Two study sites located over 2500 m above sea level (a.s.l.) in central Taiwan were selected (Figure S1). One was in the Tataka area (23°28′ N, 120°54′ E; 2550–2850 m a.s.l.) of the Experimental Forest of National Taiwan University, and another site was in the Hehuanshan area (24°09′ N, 121°17′ E; 2650–3150 m a.s.l.) of Taroko National Park. Both study sites were characterized by a temperate climate and were dominated by coniferous forests. The average annual precipitation (ca. 4000 mm) and rainy season (from April to September) are very similar at both sites.

Both study sites contained bamboo grassland (BG, *Yushania niitakayamensis*), pine woodland (PW, *Pinus taiwanensis*), and hemlock forest (HF, *Tsuga chinensis*). However, white fir forest (WFF, *Abies kawakamii*) appeared only at the Hehuanshan site and spruce forest (SF, *Picea morrisonicola*) only at the Tataka site. We set up 4 observation stations in each vegetation type for a total of 32 observation stations. These observation stations were used for collecting data on local bird community and vegetation structure. Each observation station was located in large patches of each vegetation type (>100 ha), and at least 100 m away from one another and from the edges of different vegetation types.

Based on previous studies, successional stages of the coniferous forests in the Tataka area were developing from bamboo grassland, pine woodland, hemlock forest, to spruce forest (Ding et al., 2008); and from bamboo grassland, pine woodland, white fir forest, to hemlock forest in the Hehuanshan area (Huang, 2011).

### 2.2 Transect surveys for mixed‐species bird flocks

In this study, a mixed‐species flock was defined as two or more individuals comprising of at least two species foraging and moving together in a similar direction (Morse, 1970). Fieldwork was carried out from September 2013 to February 2016. We applied the line transect method for flock survey, and at least two observers (the first author led all surveys) did the survey within each vegetation type for at least 4–5 h per trip. We conducted observations from 0600–0630 to 1100–1130 or from 1300–1330 to 1730–1800 while walking along trails in each plot. We avoided edge habitats whenever possible. When encountering a mixed‐species flock, we enumerated the number of each participating species. If two flocks fused and foraged together for more than 5 min, we considered them as a newly formed mixed flock. In total, we conducted 78 trips in the Tataka area (PW: 21 trips, BG, HF, SF: 19 trips) and 60 trips in the Hehuanshan area (BG, PW, WFF, HF: 15 trips).

After the mixed flocks disappeared, the two observers checked and discussed notes over each participating species and took the larger number of the two as the final record for each participating species. We used Garmin (GPSmap 62stc; United States) to find the location and elevation of each flock. Furthermore, we calculated flock species richness (number of bird species), flock size (number of individuals), and flock species diversity (Shannon–Wiener diversity index; Krebs, 1989) for each flock. We defined flocking frequency as the percentage of all mixed‐species flocks, across all vegetation types, where a particular species occurred (Zou et al., 2011). Only flocks persisting for 5 min or longer under observation were included in the analyses.

Most mixed‐species bird flocks were recorded in the non‐breeding season in our study sites, and flocking propensity varied with season accordingly. Since most resident species do not hold territories in the nonbreeding season, they readily become flock participants. Mixed‐species bird flocks could be divided into two major types: canopy flock and understory flock. Flamecrest (*Regulus goodfellowi*) and Taiwan Barwing (*Actinodura morrisoniana*) were the dominant nuclear species in canopy and understory flocks, respectively.

To decide whether a flock was a canopy, understorey, or mega‐flock, we relied mainly on its species composition. In this study, we defined a “mega‐flock” as a canopy or understory flock (core flock) with at least 2 additional species of the other flock type. For instance, the minimum size of a mega‐flock would consist of a canopy (or understory) flock of 2 species with 2 additional species from the other flock type. Therefore, a mega‐flock contained at least 4 bird species. Only 14 species with a flocking frequency over 5% were designated as a canopy or understory flocking species (Table 1). A bird species that was designated as understory or canopy species was based on its ecological guild (Ding et al., 2008; Liao, 2015; Table 1). Understory species typically forage on the ground or in shrubs; canopy species always forage on trees. However, bole insectivores, such as Taiwan Barwing and Eurasian Nuthatch (*Sitta europaea*), could not be clearly classified as canopy or understory species by their foraging locations in the forest. Thus, we followed a previous study, which indicated that Taiwan Barwing is a common nuclear species in understory flocks. The Eurasian Nuthatch is a common follower species in canopy flocks as indicated by a cluster analysis in Liao (2015).

**TABLE 1:** Twenty‐five bird species and one mammal species participated in 177 mixed‐species bird flocks of coniferous forests in Taiwan. Flocking frequency (%), average number of individuals in flock, and guild status of each species are presented

Common name	Scientific name^a^	Flocking frequency	Individuals in MSF	*n* ^b^	Guild^c^	Category^d^
Flamecrest	*Regulus goodfellowi*	67.8	10.1 ± 12.1	120	TI	Canopy
Coal Tit	*Periparus ater*	58.8	7.8 ± 8.9	104	TI	Canopy
Taiwan Fulvetta	*Fulvetta formosana*	53.7	5.8 ± 6.2	95	SI	Understory
Black‐throated Tit	*Aegithalos concinnus*	33.9	15.5 ± 8.9	60	TI	Canopy
Yellowish‐bellied Bush Warbler	*Horornis acanthizoides*	32.2	1.9 ± 0.9	57	SI	Understory
Green‐backed Tit	*Parus monticolus*	27.7	2.3 ± 1.1	49	TI	Canopy
White‐whiskered Laughingthrush	*Trochalopteron morrisonianum*	23.7	5.3 ± 6.0	42	SO	Understory
Eurasian Nuthatch	*Sitta europaea*	15.8	1.6 ± 0.7	28	BI	Canopy
Taiwan Barwing	*Actinodura morrisoniana*	13.0	12.2 ± 10.9	23	BI	Understory
Rufous‐capped Babbler	*Cyanoderma ruficeps*	12.4	2.1 ± 1.2	22	SI	Understory
Morrison's Fulvetta	*Alcippe morrisonia*	10.2	11.8 ± 11.1	18	SI	Understory
Collared Bush‐Robin	*Tarsiger johnstoniae*	9.0	1.3 ± 0.5	16	GI	Understory
Taiwan Yuhina	*Yuhina brunneiceps*	8.5	8.4 ± 6.8	15	TH	Canopy
Golden Parrotbill	*Suthora verreauxi*	7.3	34.6 ± 23.9	13	SI	Understory
Ferruginous Flycatcher^e^	*Muscicapa ferruginea*	4.5	2.1 ± 1.1	8	FI	Others
Eurasian Wren	*Troglodytes troglodytes*	4.0	1.3 ± 0.5	7	GI	Others
White‐browed Bush‐Robin	*Tarsiger indicus*	2.3	1.0	4	GI	Others
Steere's Liocichla	*Liocichla steerii*	2.3	2.3 ± 1.0	4	SO	Others
White‐eared Sibia	*Heterophasia auricularis*	1.7	4.3 ± 3.2	3	TH	Others
Taiwan Barbet	*Psilopogon nuchalis*	1.1	1.0	2	TH	Others
Rufous‐faced Warbler	*Abroscopus albogularis*	1.1	3.0	2	TI	Others
Eyebrowed Thrush^e^	*Turdus obscurus*	1.1	1.5 ± 0.7	2	TO	Others
Taiwan Rosefinch	*Carpodacus formosanus*	0.6	5.0	1	GH	Others
Vivid Niltava	*Niltava vivida*	0.6	1.0	1	FI	Others
Brown‐headed Thrush^e^	*Turdus chrysolaus*	0.6	1.0	1	TO	Others
Formosan striped squirrel	*Tamiops maritimus*	4.5	1.0	8	–	–

^a^ Scientific name follows Clements et al. (2019).

^b^ Sample sizes (*n*) for individuals in mixed‐species bird flocks for each species.

^c^ Ecological guild: bole insectivore, BI; fly‐catching insectivore, FI; ground herbivore, GH; ground insectivore, GI; shrub insectivore, SI; shrub omnivore, SO; tree herbivore, TH; tree insectivore, TI; tree omnivore, TO.

^d^ The bird species designated as understory or canopy species were used their ecological guild to classify them, which is used for further deciding different flock types (canopy, understory, or mega‐flock). Bole insectivores’ (BI) categories were based on a previous study of Liao (2015). Only 14 species with a flocking frequency of more than 5% were designated as canopy or understory flocking species, and the other flocking species were categorized as “Others.”

^e^ Migratory birds of Taiwan.

### 2.3 Estimating local bird communities

We conducted local bird community surveys at each station monthly from October 2013 to September 2014 by using the variable‐distance circular‐plot method (Reynolds et al., 1980). Bird counts were conducted within 3 h after local sunrise time or 3 h before local sunset time. At each observation station, we recorded the number, sex, and distance (by appearance or song if possible) of all birds heard or seen within 50 m radius during a period of 10 min. We excluded bird species that simply flew across the station and stopped the survey when it started to rain. In total, we conducted 12 monthly bird counts for each of the 32 observation stations, except 1 observation station of spruce forest with 11 counts. We then calculated bird species richness (number of bird species), bird density (number of individuals/ha), and bird species diversity (Shannon–Wiener diversity index; Krebs, 1989) for each vegetation type. The equation for calculating bird density was *D* = (*n*/*πr* ^2^ *C*) × 10^4^, where *D* was the estimated density (No./ha) of a particular species at each station, *n* was the total number of that species detected within its effective detection radius at the station, *r* was the effective detection radius (m), and *C* was the total number of bird counts (Ding et al., 2008; Reynolds et al., 1980).

### 2.4 Measurement of vegetation structure

Within each observation station, we set up a 10 m × 10 m quadrat and measured 8 vegetation attributes (Table 2) from April to June 2018. The methods in measuring these variables followed Ding et al. (2008). We used range finder and long measuring pole to measure canopy and understory heights (m) at 10 points within each quadrat and then calculated a mean for each vegetation type at both sites. Foliage cover was measured along two 10 m transects, randomly oriented transect lines that were orthogonal to each other and passed through the center of each 10 m × 10 m quadrat. We erected a carbon fiber pole at points of 1 m intervals along each transect line and counted the frequency of the pole touching a leaf, twig, or branch. The height of each contact was assigned to one of ten vertical layers (0–0.5 m, 0.5–1 m, 1–2 m, 2–4 m, 4–6 m, 6–10 m, 10–15 m, 15–20 m, 20–25 m, and >25 m). There were 20 sampling points for measuring foliage cover at each station. We calculated the foliage cover of each growth form (herb, shrub, sub‐canopy tree, and canopy tree) by averaging the number of times the pole touched a branch, twig, or leaf within vertical layers of each growth form. A 100% foliage cover meant that the pole on average touched a branch, twig, or leaf at each layer. Total foliage cover was summed from the foliage cover of all individual growth forms in the 10 vertical layers. We then applied the Shannon–Wiener diversity index (Krebs, 1989) to calculate foliage height diversity by using data from all 10 vertical layers.

**TABLE 2:** Model‐averaged estimates of predictor variables on occurrence of mega‐flock, flock species richness, and size. Models are conditional model averages of a candidate model set, consisting of all best models (ΔAICc <2), calculated from all possible model subsets

	Occurrence of mega‐flock			Species richness			Flock size		
	*β* ^a^	*p*	RVI^b^	*β* ^a^	*p*	RVI^b^	*β* ^a^	*p*	RVI^b^
Intercept	−2.60	.19		11.20	<.001		−734.83	<.01	
Forest successional gradient	**−0.69**	<.001	1.00				**−23.18**	<.01	1.00
Understory coverage							**55.03**	<.01	1.00
Foliage height diversity				**−4.01**	<.01	1.00	**753.39**	<.01	1.00
Total foliage cover	0.00	.67	0.13				**−1.43**	<.001	1.00
Bird species richness	0.16	.08	0.73	**0.13**	<.01	0.35	0.05	.97	0.52
Bird density	−0.05	.58	0.14				0.45	.67	0.48
Bird species diversity	0.83	.23	0.40	**0.75**	<.05	0.65	−9.85	.19	1.00
Candidate models		5			2			4	
Avg. conditional *r* ^2^ ^c^		.30			.13			.08	
Avg. marginal *r* ^2^ ^c^		.30			.07			.08	

^a^ β estimates; bolded values represent significant predictor variables (*p *< .05).

^b^ Relative variable importance (RVI), corresponding to the proportion of the total Akaike weight represented by all models in which the variable was included.

^c^ Average conditional and marginal *r* ^2^ were calculated across all models in the candidate subset.

### 2.5 Data analysis

Species richness and flock size among 3 flock types (canopy, understory, and mega‐flock) were compared using ANOVA for overall testing and Tukey HSD for multiple comparisons. A chi‐square test examined the homogeneity in frequencies of 3 flock types among 4 forest habitats. In addition, we used the test of standardized residuals to identify extreme high cell chi‐square values that were significantly apart from the expected value (Agresti, 1990). When the standardized residual was larger than 2 or less than −2, we considered this as a lack of fit.

We found several high correlations among different vegetation variables, and there was a lack of independence among these variables. Thus, to reduce redundancy and minimize correlation between vegetation variables, we ordinated our original vertical forest structure data by using principal component analysis (PCA) for each observation station. The principal components were interpreted using the significance of the principal component loadings. The results of the forest data ordination are shown in the Tables S1 and S2. A PCA was applied using the “prcomp” function of the stats package. The Shannon Index was run using the diversity function in the vegan package (Oksanen et al., 2020).

We ran generalized linear mixed models (glmer function, lme4 package; Bates et al., 2015) to determine the relative importance of different forest variables and local bird community characteristics on mixed‐species flock characteristics. We used a logit link function to model occurrence of mega‐flocks and an identity link function to model flock species richness, and flock size. Models contained 7 fixed‐effect predictor variables (Table 2), including both vegetation variables and local bird community characteristics. Also, we included transect ID (*n* = 6) and site (*n* = 2) as 2 random effects to account for the nonindependence of flocks observed on the same transect. To determine best models explaining flock characteristics, we used AIC values adjusted for small sample size (AICc; Burnham & Anderson, 2002) to compare models. Here, we ranked all subsets of the global model (7 fixed effects, 2 random effects; *n* = 128 models), and considered models as equivalent if their ΔAICc value was within 2 (Jones & Robinson, 2020).

We used the model.avg function (MuMIn package; Barton, 2020) to perform conditional model averaging for all response variables in the candidate model set (all models within 2 ΔAICc; Jones & Robinson, 2020; Nakagawa & Freckleton, 2011; Richards et al., 2011). Also, we evaluated the marginal and conditional *r* ^2^ values by running the r.squaredGLMM function (MuMIn package; Nakagawa & Schielzeth, 2013). We also evaluated goodness‐of‐fit using the marginal and conditional *r* ^2^ values by running the r.squaredGLMM function of the MuMIn package (Barton, 2020; Nakagawa & Schielzeth, 2013). All statistical analyses and figure plotting were performed in R 4.0.3 (R Core Team, 2020).

## 3 RESULTS

### 3.1 Composition and size of mixed‐species flocks

Twenty‐five bird species and one mammal species (Formosan striped squirrel *Tamiops maritimus*) were found participating in mixed‐species flocks (Table 1), with an average of 3.9 ± 1.6 species (range 2–11) and 29.4 ± 24.1 individuals (range 5–111) per flock (*n* = 177). The mixed‐species flocks were formed predominantly by resident bird species from our study sites. Fourteen resident bird species had a flocking frequency above 5% (Table 1). Only 3 migrant species (Ferruginous Flycatcher *Muscicapa ferruginea*, Eyebrowed Thrush *Turdus obscurus*, and Brown‐headed Thrush *Turdus chrysolaus*) were recorded in mixed‐species flocks; and their flocking frequencies and numbers were relatively low (Table 1).

**FIGURE 1:**
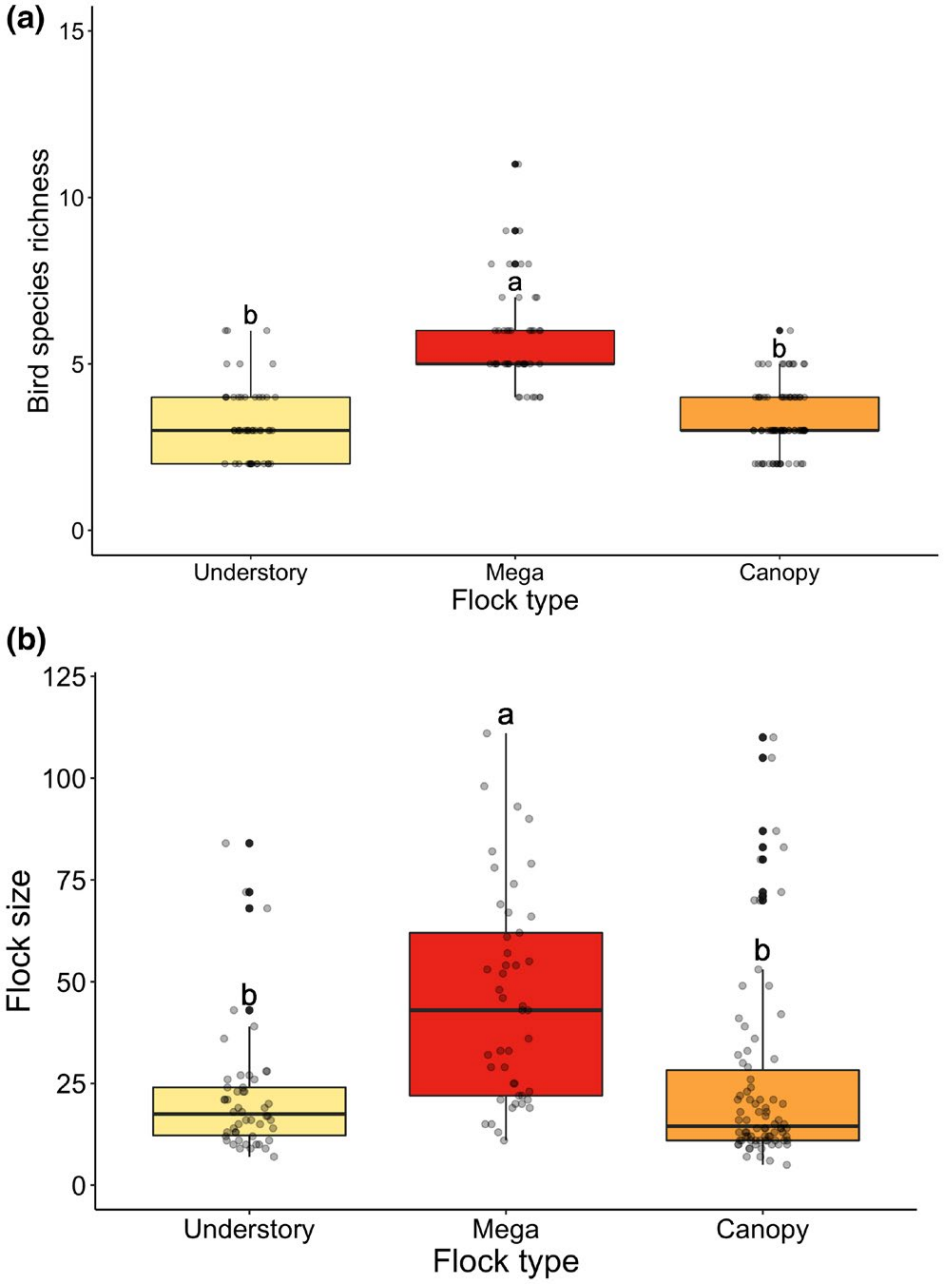
Mega‐flocks had a larger species richness (a; ANOVA, *F*_2,176_ = 76.16, *p* < .001) and flock size (b; ANOVA, *F*_2,176_ = 16.82, *p* < .001) than either canopy or understory flocks in coniferous forests of Taiwan. Different letters indicated significant differences among means based on Tukey's HSD tests

**FIGURE 2:**
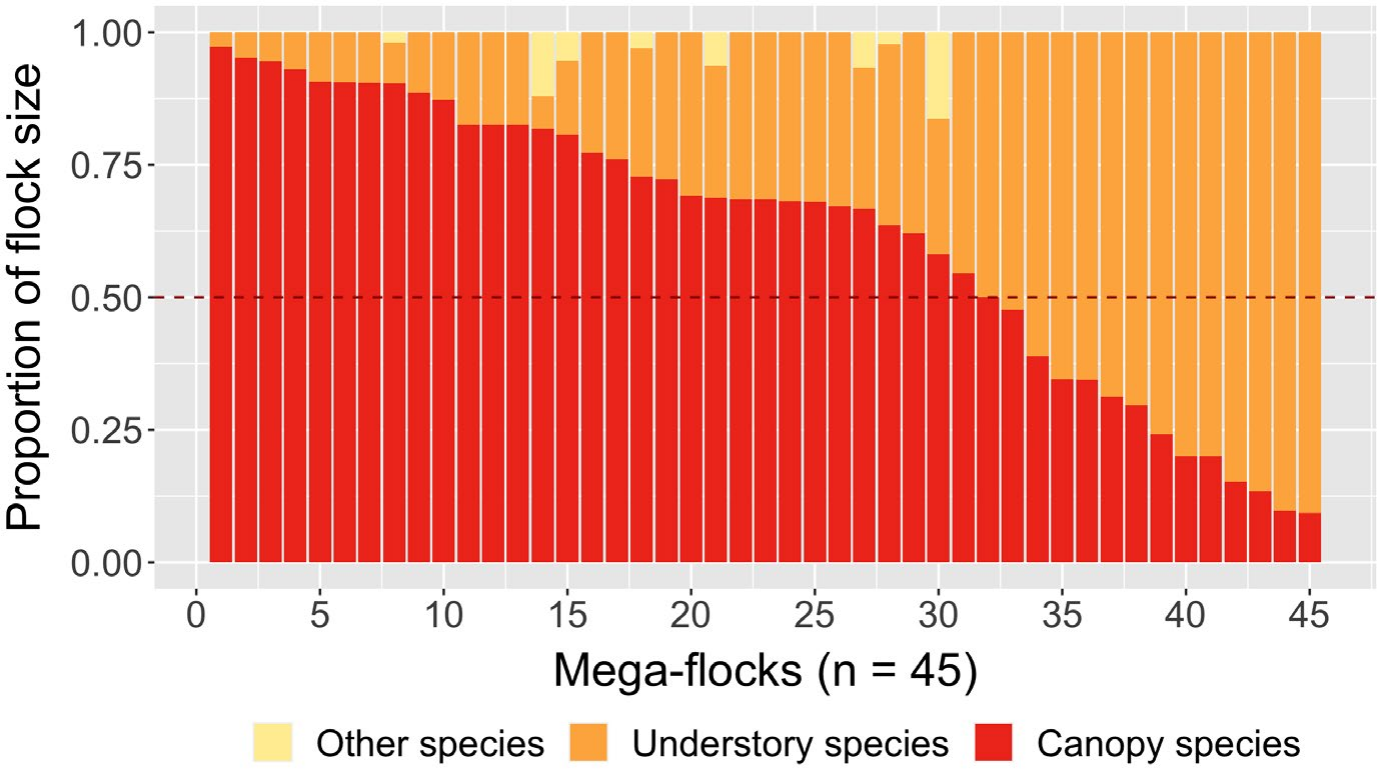
The proportions of 3 categories (canopy, understory, others) of flocking species in each mega‐flock (*n* = 45). Only the 14 species with a flocking frequency over 5% were designated as a canopy or understory flocking species (Table 1, Table S4)

Mega‐flocks usually had a more complex and larger species composition than either canopy or understory flocks. Species richness in mega‐flocks was significantly greater than that in canopy and understory flocks (mean = 5.8 vs. 3.3 and 3.2; ANOVA, *F*_2,176_ = 76.16, *p* < .001; Figure 1a), and flock size in mega‐flocks was also significantly greater than those in canopy and understory flocks (mean = 45.8 vs. 25.0 and 21.8; ANOVA, *F*_2,176_ = 16.82, *p* < .001; Figure 1b). According to our definition, 45 mega‐flocks were found in this study, and 31 of them were mainly composed of species belonging to canopy flocks (proportion of flock size >0.5; Figure 2). That is, most mega‐flocks consisted of canopy flocks with additional members from understory flocks.

### 3.2 Bird communities and mixed‐species flocks at different habitat types

**FIGURE 3:**
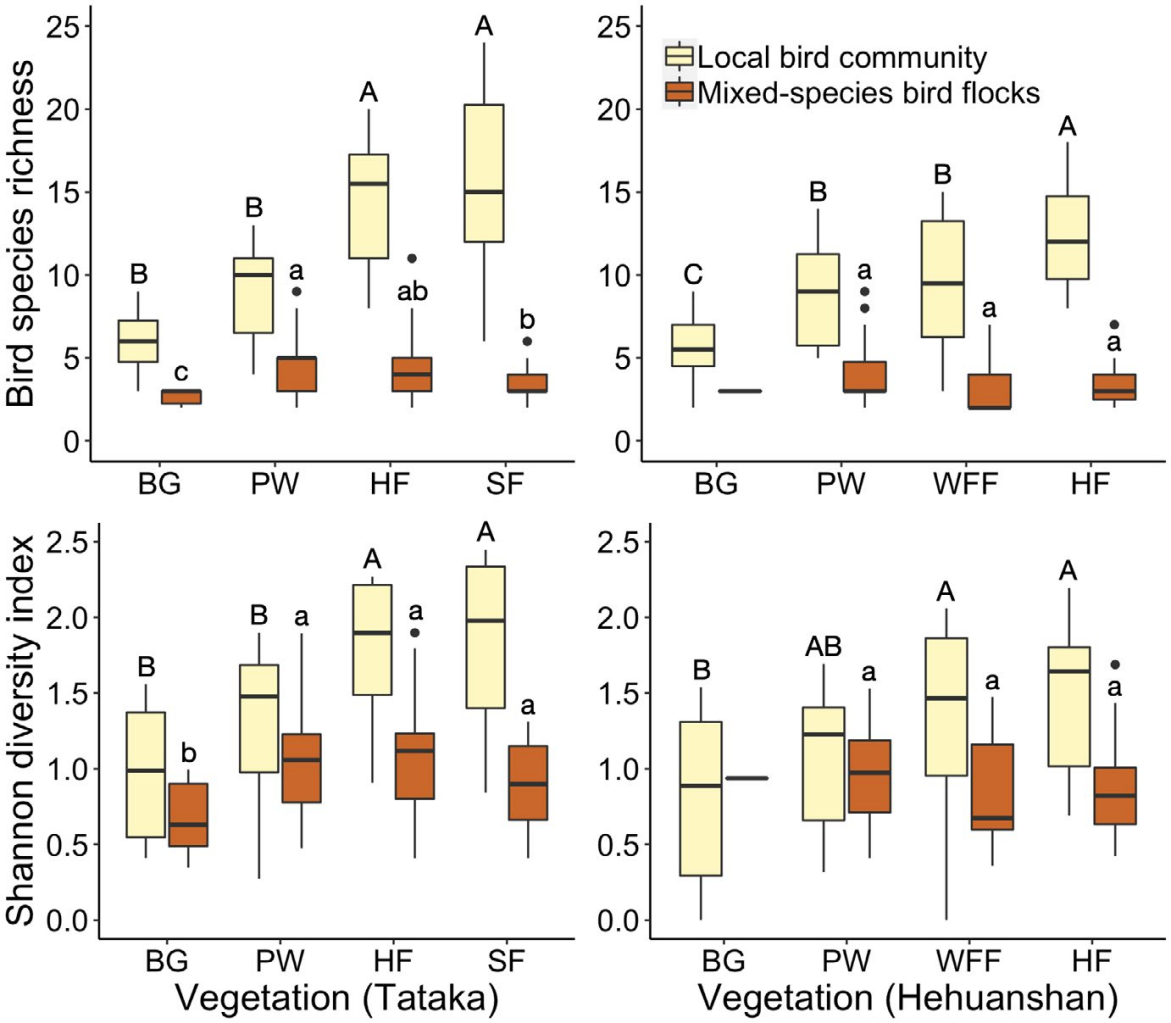
Comparison of species richness and diversity between local bird communities and mixed‐species flocks among four vegetation types of the two study sites. Different letters showed significant differences among means in Tukey HSD tests. Vegetation codes are bamboo grassland (BG), pine woodland (PW), white fir forest (WFF; only at Hehuanshan), hemlock forest (HF), and spruce forest (SF; only at Tataka)

All three bird community characteristics increased along successional stages of vegetation types, except bird density which was highest in the hemlock forest at Tataka (Table S3). Local bird species richness and diversity were lowest in bamboo grassland, and then increased through pine woodland, white fir forest, or hemlock forest, and reached the greatest values in the spruce forest (Table S3, Figure S3). However, the patterns were different in the case of mixed‐species flock variables. In the Tataka area, the greatest species richness and diversity indices of mixed‐species flocks were found in pine woodland, and then decreased gradually along the successional stages to spruce forest (Figure 3). In the Hehuanshan area, a similar trend was found for mixed‐species flocks, with greater metrics also appearing in pine woodland. However, they did not show a significant difference from those in later successional stages (Figure 3).

**FIGURE 4:**
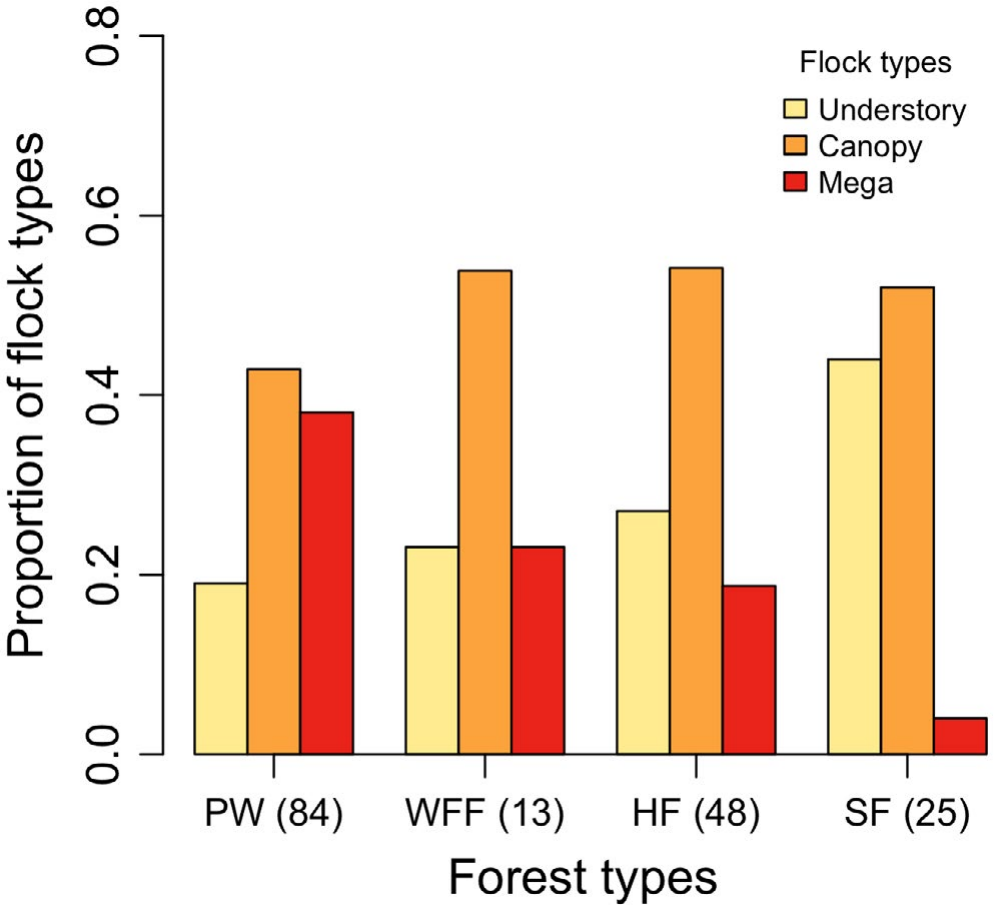
Frequencies of 3 flock types were significantly different among the 4 forest types (*χ*² =16.05, *p* < .05). Number of flocks observed is in parentheses. Vegetation codes are bamboo grassland (BG), pine woodland (PW), white fir forest (WFF), hemlock forest (HF), and spruce forest (SF)

The canopy flock was the dominant flock type in four forest types (Figure 4). The occurrence of mega‐flocks decreased along the successional stages, but understory flocks showed a reversed trend. The occurrences of the three flock types were significantly different among the four forest types (*χ*² =16.05, *p* < .05). Significant greater cell chi‐squares (or standardized residuals) were found in cells between mega‐flocks in pine woodland (4.23) or spruce forest (−4.77), and understory flocks in spruce forest (3.46). It indicated that significantly more mega‐flocks were found in pine woodland but much less in the old‐growth spruce forest. On the other hand, understory flocks were significantly more common than expected in spruce forests (Figure 4). We did not include bamboo grassland (*n* = 7) in this analysis because only understory flocks occurred in this particular habitat.

### 3.3 Vegetation structure at different habitat types

There were significant differences in all 8 physiognomic attributes among the 5 vegetation types from both study sites (ANOVA with Tukey HSD tests; Table S3). Most physiognomic attributes (canopy height, canopy cover, total foliage cover, foliage height diversity) increased with successional stages. In contrast, shrub cover peaked in earlier successional stages but decreased in later ones. Herb cover was greater in bamboo grassland and spruce forest, but lower in intermediate successional stages.

Foliage cover in bamboo grassland was found mostly below 2 m (Figure S2). The vertical profile of foliage cover was similar between pine woodland and white fir forest, and both had well‐developed vegetation layers from ground to the 10–15 m layer. The vertical profile of foliage cover in hemlock forest was similar to that of pine woodland and white fir forest except the hemlock forest had higher vegetation layer beyond 15 m. Spruce forest had a bimodal distribution of foliage cover, with the densest layers occurring above 15 m and below 1 m. The vertical profile of foliage cover was more evenly distributed in pine woodland and white fir forest, but more diverse in hemlock and spruce forest (Figure S2). As a result, the foliage height diversity increased along the successional stages, from pine woodland, to white fir forest, and to hemlock forest and spruce forest (Table S3).

### 3.4 Factors influencing flock characteristics

Our generalized linear mixed models suggested that the occurrence of mega‐flock was more strongly affected by forest structure than by local bird community characteristics (Table 2). We found a significant negative correlation between the occurrence of a mega‐flock and the forest successional gradient (PCA 1; *β* = −0.69, *p* < .001). Indices of flock species richness and flock size responded differently to our predictor variables; flock species richness was significantly affected by both forest and local bird community variables, but flock size was mainly influenced by forest vegetation structures (Table 2). Flock species richness decreased with foliage height diversity (*β* = −4.01, *p* < .01), but increased with local bird species richness (*β* = 0.13, *p* < .01) and diversity (*β* = 0.75, *p* < .05). We found significant increases in flock size in forests with greater understory coverage (PCA 2; *β* = 55.03, *p* < .01) and foliage height diversity (*β* = 753.39, *p* < .01), but a decrease in flock size with greater total foliage cover (*β* = −1.43, *p* < .001) and along successional stages of the forest (*β* = −23.18, *p* < .01).

## 4 DISCUSSION

### 4.1 The formation of mega‐flocks depends on vegetation structure

Mega‐flocks appeared to have significantly greater species richness and flock size than canopy flocks and understory flocks taken separately because they were conglomerates of the latter two flock types (Greenberg, 2000). We found that mega‐flocks occurred most frequently in pine woodland and then decreased in forests along successional stages (Table 2; Figure 4). However, a reversed trend was found for understory and canopy flocks which increased along with successional stages, in concordance with the distributional pattern of local bird communities. The occurrence of mega‐flock was negatively correlated with the forest successional gradient (PCA 1; Figure S3), which was positively correlated with canopy height and canopy cover, but negatively correlated with sub‐canopy cover (Table S2). Canopy flocks of the present study provided a major part of the local bird community in coniferous forests, and two‐thirds of mega‐flocks were composed mainly of a canopy flock with additional members from the understory flock (Figure 2). Therefore, the probability for the formation of mega‐flocks would be higher if the canopy height was lower because it will make both canopy and understory flocks to encounter with each other more readily (Poulsen, 1996). That is probably the reason why pine woodland contains more mega‐flocks than other forest types because it has the lowest canopy height. In addition, low canopy height allows sunlight to penetrate and enriches understory and sub‐canopy vegetation, and results in a better development of foliage in all layers. More evenly distributed vegetation layers would pave the way for both understory flocks to forage higher and canopy flocks to forage lower and interact with each other and form mega‐flocks.

In contrast, spruce forests have an average canopy height of 30.3 m, almost three times that of pine woodlands (11.39 m). In addition, the sub‐canopy cover in spruce forests is much lower than those in other forests, and a clear gap is created between canopy flocks and understory flocks (Figure S2). As a result, canopy and understory flocks have much greater separation between them here, and the probability to form mega‐flocks is much lower. Compared to later successional forest stages, we hereby consider the physical structure of pine woodlands as the key factor that facilitates the occurrence of mega‐flocks and, therefore, increase the abundance and diversity of mixed‐species bird flocks.

### 4.2 Effects of vegetation structure on mixed‐species flocks

Our results suggest that vegetation variables being highly correlated to local bird communities might not provide the same correlation to mixed‐species bird flocks. At our study sites, foliage height diversity increased orderly along successional stages of coniferous forests, and so did bird species richness and diversity (Table S3, Figure S3). This result supports previous findings that local bird communities are more diverse in habitats with greater foliage height diversity (Ding et al., 2008; Karr & Roth, 1971; MacArthur & MacArthur, 1961; Moss, 1978). In contrast, foliage height diversity only showed a positive effect on flock size but had no influence on flock species richness and the occurrence of mega‐flocks (Table 2). Apparently, flock complexity did not coincide well with foliage height diversity as the local bird community did. Therefore, structural heterogeneity theory could well be applied for local bird diversity (Ding et al., 2008; MacArthur & MacArthur, 1961), but not for overall mixed‐species flocks in this study. Nevertheless, when considering only understory or canopy flocks, the results are consistent with other studies and show increasing flock complexity with foliage height diversity (Colorado Z. & Rodewald, 2015; Lee et al., 2005; Mokross et al., 2014) or along successional stages of forests (Jones & Robinson, 2020; Zhang et al., 2013).

Our study observed greater flock species diversity at early and middle successional stages (pine woodland, hemlock forest) than at later successional stages (spruce forest). This result does not agree with a study conducted in Guangdong, China which showed that forests at middle and later successional stages appear to have more mixed‐species flocks and higher species diversity (Zhang et al., 2013). The authors attributed this phenomenon to the flocking propensity of regular species and the occurrence frequency of nuclear species (Huet's Fulvetta *Alcippe hueti*), which plays a vital role in such differences among vegetation types (Zhang et al., 2013). The primary nuclear species at our study sites, the Flamecrest and Coal Tit, did show a higher density at middle and later successional stages, but this seemed not to have had a strong influence on the complexity of mixed flocks (Table S4; Chen & Wang, 2008; Ding et al., 2008; Liao, 2015).

Understory coverage and foliage height diversity provided strong positive effects on flock size (Table 2). Foliage height diversity was highly correlated with local bird diversity. Of them, the two dominant canopy species, Flamecrest and Coal Tit, had a high flocking frequency and tended to enlarge flock size significantly because both are gregarious and nuclear species in mixed‐species flocks (Table 1). However, the inclusion of a dominant nuclear species in a mixed‐species flock would reduce its species diversity index simultaneously, and that is why foliage height diversity did not show any effect on flock species richness (Table 2).

The old‐growth spruce forest has greatest foliage height diversity and bird diversity, but its disruptive vegetation makes it difficult for canopy and understory flocks to meet. Even though both flock types have relatively high occurrence in spruce forests compared to other forest types, the overall complexity of mixed‐species flocks in spruce forests is indeed lower than in forests of earlier successional stages. In contrast, lower canopy height in pine woodlands might allow sunlight to penetrate and trigger the growth of subcanopy and understory vegetation. Furthermore, a better development of subcanopy and understory might even bring up primary consumers like insects and other arthropod species. Though we did not sample insects, we consider food resources as a probably additional factor that makes pine woodlands a more productive habitat for mixed‐species bird flocks. In addition, the vegetation structure in pine woodlands is less dense than that of spruce forests, and this would make flock participants more vulnerable to predators in pine woodlands even though we did not observe many predators at our study sites. However, flocking might enable them to forage more efficiently in such a more open habitat (Dolby & Grubb, Jr., 2000). These environmental factors would also facilitate the formation of mega‐flocks in pine woodlands.

### 4.3 Relationships between mixed‐species flocks and local bird communities

In this study, local bird species richness and diversity rather than bird density provides a positive effect on species richness of mixed‐species flocks (Table 2). This implies that the number of bird species rather than the number of individuals present in the local bird community contributes more to the complexity of mixed‐species flocks. Bird species richness in coniferous forests is usually much lower than in broad‐leaved forests in Taiwan (Ding et al., 2008). There are only 3.9 ± 1.6 bird species participating in mixed‐species flocks at our study sites. Therefore, adding 1 or 2 species to the flocks would provide a great change of species richness compared to flock size (29.4 ± 24.1 individuals).

Local bird communities are known to have a strong influence on mixed‐species flock diversity (Knowlton & Graham, 2011; Maldonado‐Coelho & Marini, 2004; Sridhar & Sankar, 2008). In our study, spruce forests had greatest species richness (37 species) and diversity (Table S3; Figure S3), but metrics of mixed flocks were hardly the greatest in spruce forests. In contrast, pine woodlands had the greatest mixed‐species flock metrics in our study, even though its bird community was poorer than those of forests of later successional stages. Therefore, based on our study, vegetation structure had a predominant influence on the complexity of mixed‐species flocks. We, therefore, consider the physical structure and vegetation attributes of forests as the key factors influencing the formation of mega‐flocks and those of mixed‐species bird flocks. This is the first investigation to demonstrate that vegetation characteristics governing mixed‐species bird flocks are quite different from those parameters affecting the characteristics of local bird communities.

## 5 CONCLUSION

Mixed flocking can provide both foraging and anti‐predation benefits, but the cost of participating in flocks might prevent the formation of a mega‐flock, especially in forests with multiple strata. A mega‐flock that has greater species richness and flock size than either a canopy or understory flock could contribute more to the diversity of mixed‐species bird flocks. Nevertheless, the formation of mega‐flocks is not solely dependent on the local bird community, but it is highly affected by vegetation structure of respective forests. In particular, extensive development of the mid‐story layer would bridge the gap between canopy and understory vegetation and facilitate the formation of mega‐flocks and thus the complexity of mixed‐species bird flocks. Therefore, if the costs to join flocks of different strata are reduced to a limited amount, the formation of such mega‐flocks would become possible. On the other hand, the cost of participating in flocks might deter different flock types to merge, like those in the spruce forest in this study or in tropical lowland rainforests with tall canopy and multiple vegetation strata.

## CONFLICT OF INTEREST

The authors declare no conflicts of interest.

## AUTHOR CONTRIBUTION

**Chun‐Chieh Liao:** Conceptualization (equal); Data curation (lead); Formal analysis (lead); Investigation (lead); Methodology (equal); Project administration (equal); Visualization (lead); Writing – original draft (equal). **Tzung‐Su Ding:** Conceptualization (equal); Formal analysis (equal); Funding acquisition (equal); Methodology (equal); Project administration (equal); Supervision (lead); Writing – original draft (supporting); Writing – review & editing (supporting). **Chao‐Chieh Chen:** Conceptualization (equal); Funding acquisition (equal); Methodology (equal); Project administration (equal); Writing – original draft (equal); Writing – review & editing (lead).

## Supporting information

Appendix S1

## Data Availability

Data are uploaded to Dryad digital repository https://doi.org/10.5061/dryad.9zw3r22fw.
