# Supplementary material for: The formation of “mega‐flocks” depends on vegetation structure in montane coniferous forests of Taiwan
Source: Ecol Evol. 2022 Feb 18;12(2):e8608. doi: 10.1002/ece3.8608 (PMC8855335; doi:10.1002/ece3.8608)
Supplement: Supplementary file 1 — Appendix S1 [file ECE3-12-e8608-s001.docx]

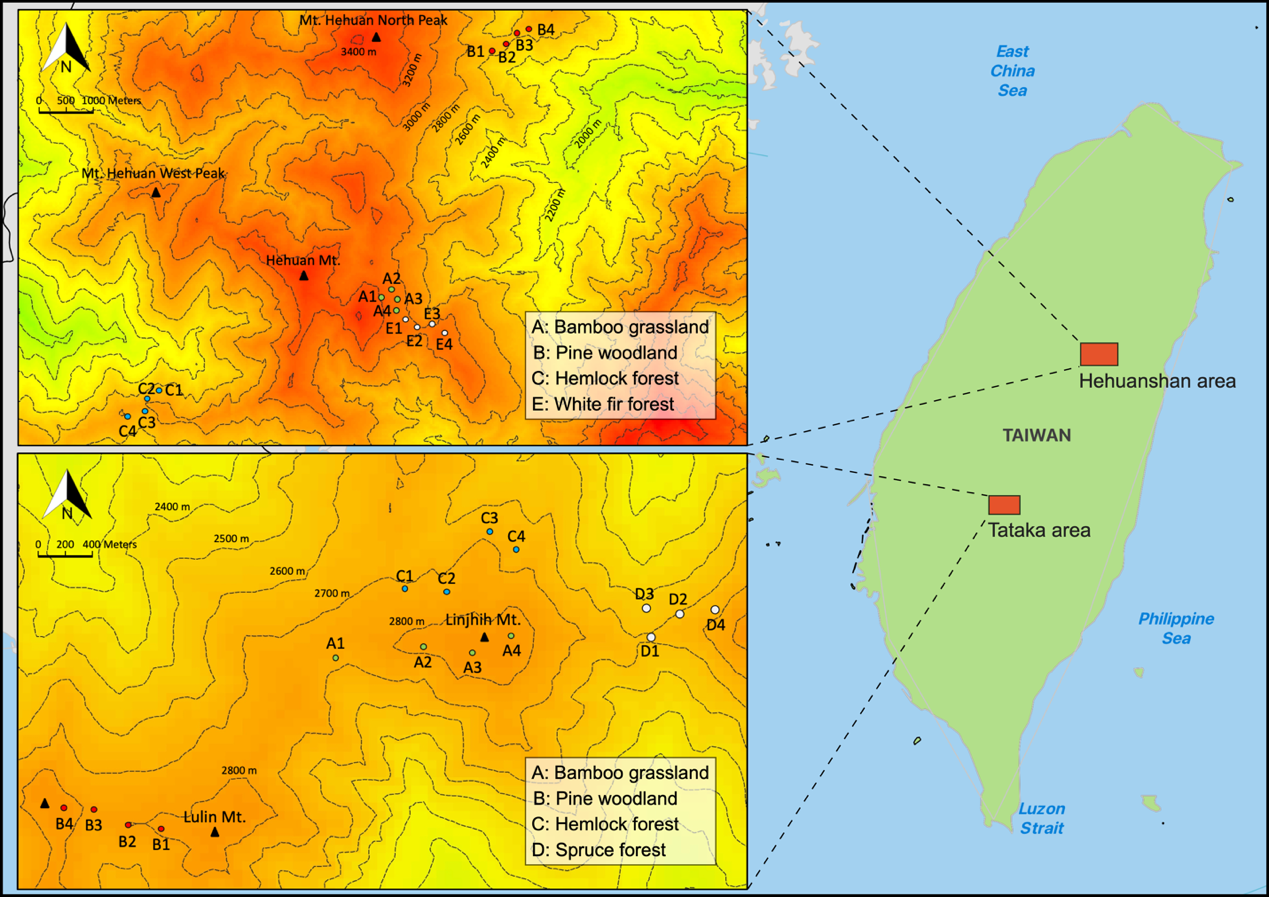


Appendix figure 1. Map of study sites, both located over 2,500 m above sea level in central Taiwan. One was in the Tataka area of the Experimental Forest of National Taiwan University, and another site was in the Hehuanshan area of Taroko National Park. Insets showed detailed allocation of observation stations within two sites.

(a)


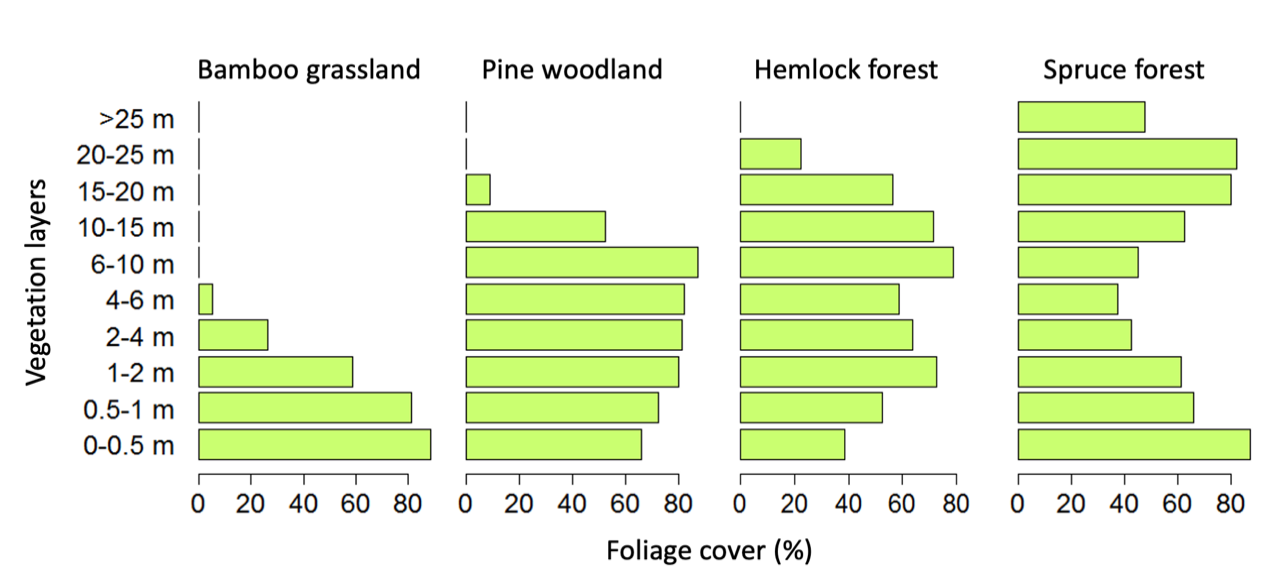


(b)


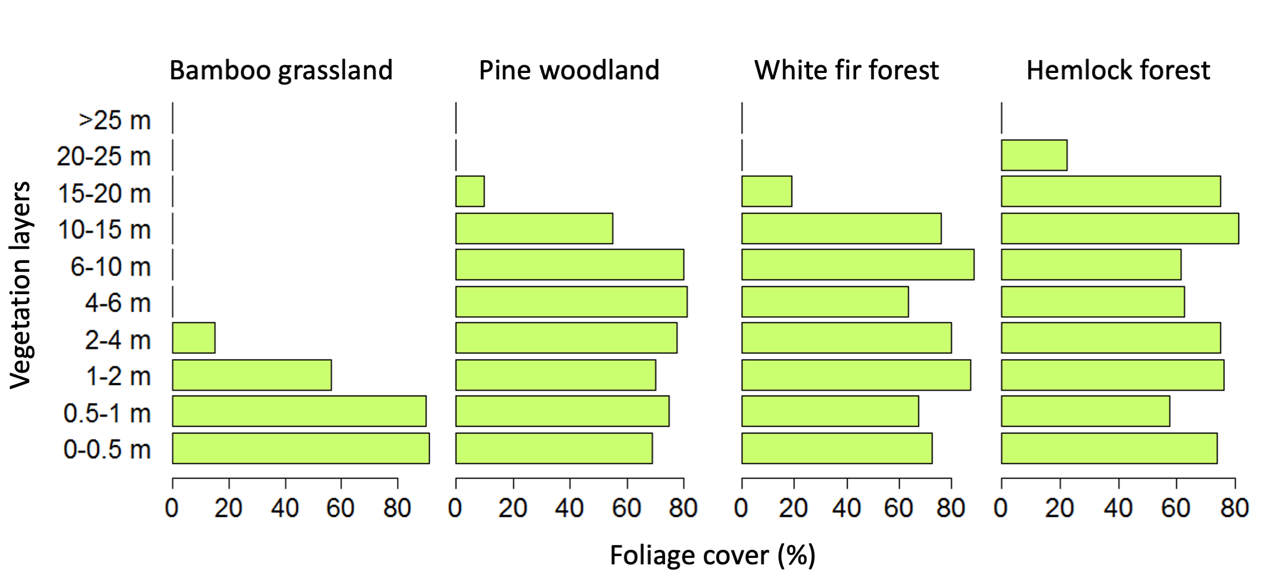


Appendix figure 2. The vertical profile of foliage covers in different vegetation types of the two study sites: (a) Tataka area and (b) Hehuanshan area.


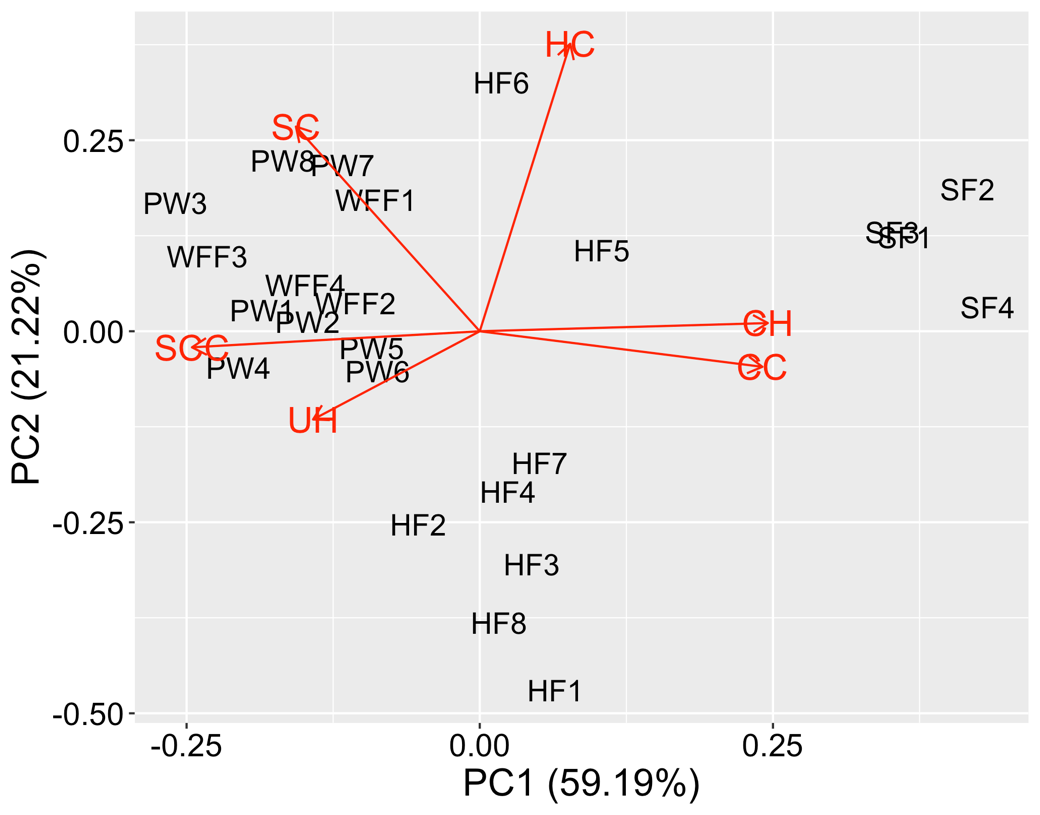


Appendix figure 3. Biplot of first 2 principal component axes of the ordinations of the vertical foliage structure. Percentages in axis labels indicate percentage of total variance explained by each axis. We interpret PCA 1 as a measure of forest successional gradient; high negative values are associated with former successional stage of forests (e.g., Pine woodland PW; White fir forest WFF). PCA 2 is interpreted as a measure of understory coverage, with higher values associated with greater coverage of understory vegetation.

Appendix table 1. Eigenvalues and proportion of variance explained of the principal component analysis of vertical foliage structure. The first and second axes (named) were retained for further GLMM analyses.

| PC Axis | Eigenvalue | Proportion of variance explained | Cumulative proportion of variance explained |
| --- | --- | --- | --- |
| Forest successional gradient | 3.551 | 0.592 | 0.592 |
| Understory coverage | 1.273 | 0.212 | 0.804 |
| 3 | 0.759 | 0.126 | 0.931 |
| 4 | 0.334 | 0.056 | 0.986 |
| 5 | 0.056 | 0.009 | 0.996 |
| 6 | 0.027 | 0.004 | 1.000 |

Appendix table 2. Loadings of the vertical foliage structure class principal component axes. Rows represent 6 vegetation variables measured for each observation station (n = 24 stations, 4 forest types). Columns represent the principal component axes; first 2 principal component axes (PCA1 was named as forest successional gradient; PCA2 was named as understory coverage) were retained for further GLMM analyses.

| Variable | PCA 1 ^a^ | PCA 2 ^a^ | PCA 3 | PCA 4 | PCA 5 | PCA 6 |
| --- | --- | --- | --- | --- | --- | --- |
| Understory height | -0.297 | -0.241 | 0.882 | 0.258 | 0.086 | -0.037 |
| Canopy height | **0.513** | 0.022 | 0.219 | -0.169 | -0.241 | -0.776 |
| Herb cover | 0.161 | **0.786** | 0.107 | 0.582 | -0.062 | 0.051 |
| Shrub cover | -0.327 | **0.559** | 0.256 | -0.716 | -0.020 | 0.035 |
| Sub-canopy cover | **-0.512** | -0.044 | -0.158 | 0.158 | -0.811 | -0.167 |
| Canopy cover | **0.503** | -0.097 | 0.268 | -0.166 | -0.522 | 0.604 |

^a^ Bolded values represent significant for the purposes of axis interpretation (loadings of > 0.3, or < -0.3)

Appendix table 3. Habitat attributes and local bird community characteristics (mean ± S.D.) in each of the eight plots^1^

| Parameter | Bamboo grassland | | Pine woodland | | White fir forest^2^ | Hemlock forest | | Spruce forest^2^ |
| --- | --- | --- | --- | --- | --- | --- | --- | --- |
|  | Tataka | Hehuanshan | Tataka | Hehuanshan | Hehuanshan | Tataka | Hehuanshan | Tataka |
| Local bird community characteristic | |  |  |  |  |  |  |  |
| Bird species richness | 5.92 ± 1.83^c^ | 5.50 ± 2.07^c^ | 8.92 ± 3.03^bc^ | 8.83 ± 3.27^bc^ | 9.25 ± 4.27^bc^ | 14.42 ± 3.85^a^ | 12.33 ± 3.55^ab^ | 16.00 ± 5.38^a^ |
| Bird density (No./ha) | 1.49 ± 0.40^d^ | 1.21 ± 0.19^d^ | 3.56 ± 0.51^cd^ | 2.83 ± 0.42^cd^ | 6.37 ± 2.25^abc^ | 10.02 ± 3.45^a^ | 5.99 ± 1.88^bc^ | 8.12 ± 1.50^ab^ |
| Bird species diversity | 0.96 ± 0.44^d^ | 0.81 ± 0.57^d^ | 1.29 ± 0.53^bc^ | 1.05 ± 0.47^cd^ | 1.33 ± 0.67^bc^ | 1.78 ± 0.47^a^ | 1.45 ± 0.53^b^ | 1.81 ± 0.56^a^ |
| Total bird species | 17 | 11 | 24 | 28 | 20 | 32 | 33 | 37 |
|  |  |  |  |  |  |  |  |  |
| Physiognomic attribute |  |  |  |  |  |  |  |  |
| Canopy height (m) | 0.00 ± 0.00^e^ | 0.00 ± 0.00^e^ | 12.05 ± 0.74^cd^ | 10.73 ± 1.72^d^ | 13.6 ± 1.42^c^ | 17.53 ± 0.45^b^ | 18.50 ± 0.91^b^ | 30.30 ± 0.34^a^ |
| Understory height (m) | 1.34 ± 0.39^bc^ | 1.33 ± 0.34^bc^ | 2.73 ± 0.04^a^ | 1.22 ± 0.16^c^ | 2.83 ± 0.73^a^ | 2.28 ± 0.29^ab^ | 2.69 ± 0.28^a^ | 1.01 ± 0.16^c^ |
| Herb cover (%) | 88.75 ± 6.29^a^ | 91.25 ± 2.50^a^ | 66.25 ± 4.79^ab^ | 68.75 ± 18.87^a^ | 72.50 ± 15.55^a^ | 38.75 ± 4.79^b^ | 73.75 ± 23.94^a^ | 87.50 ± 6.45^a^ |
| Shrub cover (%) | 171.25 ± 41.31^a^ | 161.25 ± 27.20^a^ | 152.50 ± 11.90^a^ | 145.00 ± 0.00^a^ | 155.00 ± 10.80^a^ | 125.00 ± 13.54^a^ | 133.75 ± 20.56^a^ | 127.50 ± 12.58^a^ |
| Sub-canopy cover (%) | 0.00 ± 0.00^d^ | 0.00 ± 0.00^d^ | 251.25 ± 11.09^a^ | 238.75 ± 12.50^a^ | 232.50 ± 14.43^a^ | 201.25 ± 14.36^b^ | 198.75 ± 18.87^b^ | 125.00 ± 17.80^c^ |
| Canopy cover (%) | 0.00 ± 0.00^e^ | 0.00 ± 0.00^e^ | 61.25 ± 22.87^d^ | 65.00 ± 46.37^d^ | 95.00 ± 33.91^cd^ | 150.00 ± 22.73^bc^ | 178.75 ± 10.31^b^ | 272.50 ± 9.57^a^ |
| Total foliage cover (%) | 260.00 ± 33.35^d^ | 252.50 ± 24.11^d^ | 531.25 ± 24.08^bc^ | 517.50 ± 30.10^c^ | 555.00 ± 17.68^abc^ | 515.00 ± 6.12^c^ | 585.00 ± 25.74^ab^ | 612.50 ± 12.99^a^ |
| Foliage height diversity | 1.33 ± 0.10^d^ | 1.22 ± 0.06^d^ | 1.97 ± 0.04^c^ | 1.94 ± 0.10^c^ | 1.99 ± 0.05^bc^ | 2.14 ± 0.02^ab^ | 2.15 ± 0.04^ab^ | 2.26 ± 0.01^a^ |

^1^ For all variables except shrub cover, significant differences were found among vegetation types (ANOVA, Tukey HSD test). The values that are not significantly different (*p* > 0.05) are indicated by shared superscript letters.

^2^ White fir forest only appeared at the Hehuanshan site and spruce forest only at the Tataka site.

Appendix table 4. The average density (mean ± S.D.) (No./ha) of 14 flocking species (flocking frequency > 5%) in each of the 8 plots.

| Common name | Flock type | Tataka area | | | |  | Hehuanshan area | | | |
| --- | --- | --- | --- | --- | --- | --- | --- | --- | --- | --- |
|  |  | BG | PW | HF | SF |  | BG | PW | WFF | HF |
| Flamecrest | Canopy | 0.07±0.26 | 3.09±1.22 | 11.05±3.91 | 7.96±4.22 |  | 0.15±0.51 | 2.28±2.37 | 7.81±3.61 | 5.97±2.53 |
| Coal Tit | Canopy |  | 0.16±0.30 | 1.00±0.61 | 0.83±0.56 |  |  | 0.57±0.48 | 0.24±0.27 | 0.72±0.51 |
| Black-throated Tit | Canopy | 0.15±0.51 |  | 0.66±1.37 |  |  |  | 1.03±1.88 |  | 0.44±1.28 |
| Green-backed Tit | Canopy |  | 0.03±0.09 | 0.32±0.41 | 0.16±0.32 |  |  | 0.03±0.09 |  | 0.05±0.12 |
| Eurasian Nuthatch | Canopy |  |  |  |  |  |  | 0.07±0.26 |  | 0.22±0.77 |
| Taiwan Yuhina | Canopy | 0.06±0.15 | 0.34±0.42 | 1.08±0.91 | 1.19±0.96 |  |  | 0.57±0.58 | 0.06±0.15 | 1.79±1.26 |
| Taiwan Fulvetta | Understory | 2.98±1.27 | 3.15±1.13 | 3.94±1.55 | 2.74±1.05 |  | 1.08±0.67 | 1.82±1.17 | 2.82±1.41 | 2.45±1.13 |
| Yellowish-bellied Bush-Warbler | Understory | 0.92±0.62 | 0.83±0.51 | 1.27±0.95 | 0.41±0.38 |  | 0.68±0.64 | 0.29±0.39 | 0.94±0.80 | 0.85±0.74 |
| White-whiskered Laughingthrush | Understory | 0.30±0.19 | 0.37±0.24 | 0.34±0.18 | 0.34±0.18 |  | 0.28±0.13 | 0.29±0.24 | 0.36±0.17 | 0.26±0.12 |
| Taiwan Barwing | Understory |  |  | 0.48±0.74 | 0.82±0.82 |  |  | 0.19±0.44 |  | 0.58±0.72 |
| Rufous-capped Babbler | Understory |  | 0.04±0.08 | 0.12±0.14 | 0.21±0.20 |  |  | 0.05±0.08 |  | 0.03±0.08 |
| Morrison’s Fulvetta | Understory |  | 0.08±0.29 | 1.49±2.77 | 1.57±1.60 |  |  |  |  | 0.45±0.76 |
| Collared Bush-Robin | Understory | 0.27±0.35 | 0.34±0.37 | 0.74±0.34 | 0.21±0.25 |  | 0.77±0.72 | 0.27±0.33 | 0.27±0.33 | 0.34±0.32 |
| Golden Parrotbill | Understory |  | 0.81±1.90 | 0.88±2.35 | 0.22±0.77 |  | 0.07±0.26 |  | 0.59±1.32 |  |
